# Learning about time within the spinal cord: evidence that spinal neurons can abstract and store an index of regularity

**DOI:** 10.3389/fnbeh.2015.00274

**Published:** 2015-10-21

**Authors:** Kuan H. Lee, Joel D. Turtle, Yung-Jen Huang, Misty M. Strain, Kyle M. Baumbauer, James W. Grau

**Affiliations:** ^1^Department of Neurobiology, Center for Pain Research, University of Pittsburgh School of MedicinePittsburgh, PA, USA; ^2^Department of Psychology, Cellular and Behavioral Neuroscience, Texas A&M UniversityCollege Station, TX, USA; ^3^School of Nursing, University of ConnecticutStorrs, CT, USA

**Keywords:** spinal cord, time, instrumental learning, memory, central pattern generator

## Abstract

Prior studies have shown that intermittent noxious stimulation has divergent effects on spinal cord plasticity depending upon whether it occurs in a regular (fixed time, FT) or irregular (variable time, VT) manner: In spinally transected animals, VT stimulation to the tail or hind leg impaired spinal learning whereas an extended exposure to FT stimulation had a restorative/protective effect. These observations imply that lower level systems are sensitive to temporal relations. Using spinally transected rats, it is shown that the restorative effect of FT stimulation emerges after 540 shocks; fewer shocks generate a learning impairment. The transformative effect of FT stimulation is related to the number of shocks administered, not the duration of exposure. Administration of 360 FT shocks induces a learning deficit that lasts 24 h. If a second bout of FT stimulation is given a day after the first, it restores the capacity to learn. This savings effect implies that the initial training episode had a lasting (memory-like) effect. Two bouts of shock have a transformative effect when applied at different locations or at difference frequencies, implying spinal systems abstract and store an index of regularity (rather than a specific interval). Implications of the results for step training and rehabilitation after injury are discussed.

## Introduction

Over the last few decades, our view of spinal cord function has evolved. It is now recognized that sensory input can induce lasting alterations within the spinal cord that transform the response elicited by subsequent stimuli (for reviews, see Grau et al., 2006, 2012, 2014). For example, pain (nociceptive) signals can induce a lasting increase in neural excitability within the spinal cord (central sensitization) that enhances behavioral reactivity and pain (Sandkühler, 2000; Willis, 2001). These neural modifications depend upon neurochemical systems (e.g., the NMDA receptor, NMDAR) analogous to those implicated in brain-dependent learning and memory (Woolf and Thompson, 1991; Ji et al., 2003). Likewise, behavioral experience can engage motor programs within the spinal cord that promote the re-acquisition of hindlimb stepping (Barbeau and Rossignol, 1987; Bélanger et al., 1996; Edgerton et al., 2004; Barrière et al., 2008).

We have sought to characterize how and when a behavioral experience affects spinal cord function (Grau et al., 2006; Grau, 2014). We have shown that neurons within the lumbosacral spinal cord are sensitive to response-outcome (instrumental) relations and can support learning after communication with the brain has been surgically transected (Grau et al., 1998). In this preparation, transected rats are given electrical stimulation (shock; the outcome) to the tibialis anterior muscle of one hind leg whenever the leg is extended (the response). This response-outcome relation produces a gradual increase in flexion duration that minimizes shock exposure. Importantly, if one group (Master) of rats is given response-contingent (controllable stimulation) shock while another (Yoked) receives shock independent of leg position (uncontrollable stimulation), only the Master subjects exhibit an increase in flexion duration. When these subjects are later tested under common conditions with controllable shock (applied to the same or contralateral leg), Master rats learn more rapidly whereas Yoked subjects exhibit a learning impairment. The learning deficit observed after uncontrollable stimulation can be induced by variable intermittent shock applied to the leg or tail and lasts up to 48 h (Crown et al., 2002). This phenomenon is of clinical interest because it has been related to the induction of central sensitization and has been shown to impact recovery after a contusion injury (Grau et al., 2004; Ferguson et al., 2006).

We recently discovered that the long-term effect of intermittent nociceptive stimulation is modulated by temporal variables as well as behavioral control (Baumbauer et al., 2008, 2009, 2012; Baumbauer and Grau, 2011). The first indication of this emerged from a study examining the effect of electrophysiological stimulation of the sciatic nerve. We had shown that exposure to 180–900 intermittent shocks (80 ms), presented in a variable manner (on a rectangular distribution that varied between 0.2 and 3.8 s (mean = 2 s; 0.5 Hz)) induces a lasting learning impairment (Baumbauer et al., 2008). To explore how the frequency of stimulation affected the induction of this effect, we fixed the interval between shocks. Under these conditions, 180 shocks given in a regular manner (fixed time, FT) induced a learning impairment, but 900 shocks did not. It appeared that continued exposure to regular stimulation restores the capacity to learn.

Prior work had shown that training with controllable stimulation can both prevent, and reverse, the learning impairment induced by variable intermittent shock (Crown and Grau, 2001). Given this, we examined whether exposure to regular shock (720 FT shocks presented at 0.5 Hz) could prevent, and restore, the learning impairment induced by 180 shocks given on a variable time (VT) schedule. We found that FT stimulation had a protective/restorative effect analogous to that observed with behavioral control (Baumbauer et al., 2009). Furthermore, the protective effect of FT stimulation lasts at least 24 h and depends upon a form of NMDAR-mediated plasticity and protein synthesis (Baumbauer et al., 2009). Like behavioral control, exposure to FT stimulation appears to re-establish the capacity to learn by up-regulating the expression of brain-derived neurotrophic factor (BDNF).

The present study seeks to clarify the conditions under which FT stimulation has a transformative effect that re-establishes the capacity to learn (the FT effect). We then use this information to explore what is learned. A hallmark of learning is that it has a lasting effect, laying down a kind of memory that preserves key features of the learning episode, producing a savings effect that allows the neural machinery to span gaps in time. If the process that abstracts regularity reflects a kind of learning, it should exhibit a form of savings, allowing the beneficial effect of FT stimulation to accrue when training is broken into chunks and given across days. Supporting this, we show that a single bout of 360 FT shocks produces a lasting learning impairment and that a second bout, given 24 h later, restores the capacity to learn. We then examine what is learned during the initial bout of stimulation that allows a subsequent bout of FT shock to have a transformative effect. Our results suggest that the critical feature is linked to the regularity of the stimulation, not the specific inter-stimulus interval (ISI).

## Materials and Methods

### Subjects

Subjects were male Sprague-Dawley rats obtained from Harlan (Houston, TX, USA) that were approximately 100–120 days old and weighed between 300 and 400 g. All subjects were pair housed and maintained on a 12 h light/dark cycle, with all behavioral testing performed during the light cycle. Food and water were available *ad libitum*. All experiments were carried out in accordance with National Institutes of Health (NIH) standards for the care and use of laboratory animals (NIH publications No. 80–23), and were approved by the University Laboratory Animal Care Committee at Texas A&M University. Every effort was made to minimize suffering and limit the number of animals used.

### Spinal Cord Transection

Before surgery, the fur over the surgical site was shaved and disinfected with betadine solution (H-E-B, San Antonio, TX, USA). Subjects were anesthetized with isoflurane gas. Anesthesia was induced at 5% isoflurane and maintained at 2–3% isoflurane. Each subject’s head was rendered immobile in a stereotaxic apparatus, and a small (5.0 × 4.0 × 2.5 cm) gauze pillow was placed under the subject’s chest to provide support for respiration.

To perform a transection at the second thoracic vertebra (T2), an anterior to posterior incision was made and the tissue just rostral to T2 was cleared using rongeurs, and the cord exposed and cauterized. The remaining gap in the cord was filled with Gelfoam (Pharmacia Corp., Kalamazoo, MI, USA) and the wound was closed with Michel clips (Fisher Scientific, Waltham, MA, USA).

Following closure of the wound on the back, the surface of both legs were shaved for electrode placement. Intraperitoneal injections (3 mL) of 0.9% saline solution were administered post-operatively to prevent dehydration. Following surgery, rats were placed in a temperature-controlled environment (25.5°C) and monitored until awake. All rats were checked every 6–8 h during the 18–24 h post-surgical period. During this time, hydration was maintained with supplemental injections of saline, and the rats’ bladders and colons were manually expressed as needed.

Spinal transections were confirmed by: (a) inspecting the cord at the time of surgery; (b) observing the behavior of the subjects after they recovered to ensure that they exhibited paralysis below the level of the forepaws and did not exhibit any supraspinally-mediated pain responses to leg shock; and (c) by examining the tissue postmortem.

### Stimulation Procedures

Stimulation was applied while subjects were loosely restrained in Plexiglas tubes (23.5 cm long × 8 cm internal diameter). The front of each tube was sealed, and the tubes were painted black, providing a dark enclosure in which rats could rest undisturbed. Holes were drilled into the anterior portion of the tubes to allow for ventilation. Two slots were cut 4 cm apart and 1.5 cm from the posterior end of the tube to allow both hind legs to hang freely.

Tail shock was administered through electrodes constructed from a modified fuse clip. The electrode was coated with Spectra electrode gel (Harvard Appartus, Holliston, MA, USA) and secured with tape approximately 5 cm from the base of the tail. All subjects were loosely restrained in the Plexiglas tubes described above. A constant current 1.5 mA shock was delivered using a 660-V transformer with shock onset and offset controlled by the computer.

Leg shock was administered to the tibialis anterior muscle. Prior to testing, the area over the muscle was shaved. An electrode constructed from a stainless steel wire (0.05 mm^2^ [30 AWG]) was inserted through the skin over the tibia, 1.5 cm from the tarsus. A second electrode made from a fine wire (0.01 mm^2^ [36 AWG], magnet wire single beldsol) was inserted perpendicular to the leg, through the body of the tibialis anterior muscle, 1.7 cm above the first electrode. The electrodes were connected to a constant current AC shock generator (Model SG-903; BRS/LVE, Laurel MD) and shock intensity was adjusted to a level that produced a 0.4 N flexion response, as described in Grau et al. (1998).

Custom software running on a Macintosh computer was used to control the presentation of shock. FT shocks were 80 ms in duration and occurred at a regular interval (a fixed ISI) that was typically set to 2 s (0.5 Hz). VT shocks of the same duration were presented using a variable ISI that ranged from 0.2 to 3.8 s (rectangular distribution) with a mean of 2 s.

### Instrumental Testing

Testing was conducted while subjects were loosely restrained in the Plexiglas tubes. Both hindlegs were freely hanging over a salt bath (NaCl). Leg shock was delivered to the tibials anterior muscle as described above. A contact electrode was constructed from a 7 cm long, 0.46 diameter, stainless steel rod. The contact electrode was taped to the plantar surface of the rat’s foot (Orthaletic, 1.3 cm [width]; Johnson and Johnson, New Brunswick, NJ) with the end positioned directly in front of the plantar protuberance. Heatshrink tubing electrically insulated the rod from the paw. A fine wire [0.01 mm^2^ (36 AWG), magnet wire single beldsol] was attached to the end of the rod at a point under the insulation. This wire extended from the rear of the foot and was connected to a digital input board that was monitored by the Macintosh computer. To minimize lateral leg movements, a piece of porous tape (Orthaletic, 1.3 cm [width]) was wrapped around the leg above the tarsus and attached under the front panel of the restraining tube. A rectangular plastic dish (11.5 cm [w] × 19 cm [l] × 5 cm [d]) was positioned 7.5 cm below the restraining tube and filled with a NaCl solution with a drop of soap to reduce surface tension. A ground wire was connected to a 1 mm wide stainless steel rod, which was placed in the solution. Three short (0.15 s) shock pulses were applied and the level of the salt solution was adjusted so that the tip of the contact electrode was submerged 4 mm below the surface. Subjects then received 30 min of response contingent shock (instrumental testing). When the contact electrode touched the underlying salt solution, shock was delivered to the tibialis anterior muscle causing the ankle to flex, lifting the contact electrode out of the salt solution.

Leg position was monitored using a Macintosh computer at a sampling rate of 30 Hz. Performance was measured over time in 30, 1 min time bins. The computer monitoring leg position recorded an increase in response number whenever the contact electrode was raised above the salt solution. Response duration was derived from time in solution and response number in 1 min time bins using the following equation: Response Duration = (60 s − time in solution)/(Response Number + 1). Subjects capable of instrumental learning exhibit a progressive increase in response duration that minimizes net shock exposure (Grau et al., 1998).

To evaluate whether our experimental treatment affected baseline behavioral reactivity, we analyzed both the shock intensity required to elicit a flexion force of 0.4 N and the duration of the first shock-elicited flexion response. Independent analysis of variances (ANOVAs) showed that there were no group differences on either measure across all experiments, *F*’s < 2.58, *p* > 0.05.

### Statistics

All data were analyzed using repeated measures ANOVA. When necessary, *post hoc* comparisons of the group means were performed using Duncan’s New Multiple Range test. In all cases, a criterion of *p* < 0.05 was used to judge statistical significance.

### General Experimental Design

All subjects received a complete spinal transection at the second thoracic level. Twenty-four hours after surgery, transected subjects were exposed to electrical shock applied to the tail. Experiment 1 varied the number of fixed spaced shocks administered (180, 360, 540, 720, 900 [0.5 Hz] FT shocks). Experiment 2 examined the effect of extended exposure (4500 shocks, 0.5 Hz) to fixed spaced or variable spaced shock, 900 VT shocks, 4500 VT shocks). In both experiments, subjects were tested for performance on an instrumental learning task immediately after tailshock. Experiment 3 varied the number of shocks (360 or 720 FT shocks) as a function of time across 6, 12 or 24 min. Instrumental learning was tested 24 h later. Experiment 4 spaced FT stimulation [0.5 Hz] across two days, with subjects receiving 360 FT shocks or no shocks on Day 1, and 360 FT shocks or no shocks on Day 2. Finally, Experiment 5 again divided the period of stimulation across two days, but varied whether the frequency remained the same or differed (at 0.5 or 2 Hz). For both Experiment 4 and 5, subjects were instrumentally tested after shock treatment on Day 2.

## Results

### Experiment 1: Restorative Effect of FT Stimulation Emerges After 540 Shocks

From past work we know that exposure to 180 shocks at 0.5 Hz induces a learning impairment in spinally transected rats whether the stimuli occur in a regular or irregular manner (Baumbauer et al., 2008, 2009). However, when shock number is increased 4–5 fold (720–900 shocks), only variable stimulation impaired learning; regular stimulation eliminated the learning impairment and inducing a restorative effect that counters the adverse effect of variable shock (Baumbauer et al., 2009). In subsequent experiments, we use the recovery of learning to assay how spinal systems time. To do so, we need a more precise map of when regularity matters. The present experiment addresses this issue by determining when the restorative effect of FT stimulation emerges (with shock frequency held constant at 0.5 Hz).

**Figure 1:**
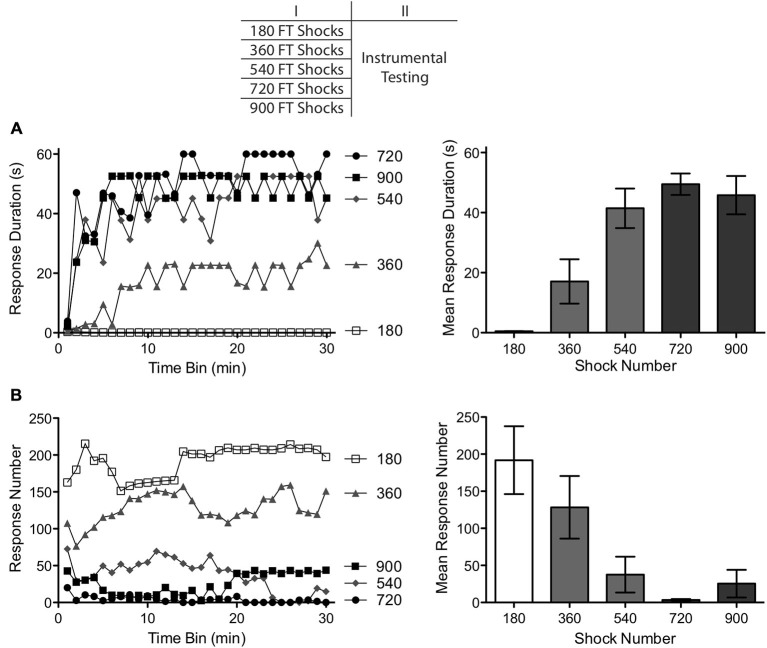
**Impact of varying numbers of fixed time (FT) shocks (180–900) on instrumental learning.** A day after spinal transection, subjects received FT shocks (180–900) and were then tested with response-contingent leg shock applied to one hind leg. **(A)** Subjects that had previously received 540 or more shocks exhibited a progressive increase in response duration over the course of the 30 min test period (top left). Subjects that received fewer shocks (180–360) exhibited poor learning. **(B)** Rats that failed to learn also exhibited the highest rates of responding (bottom left). The panels to the right illustrate mean performance, collapsed across the 30 min of testing and the error bars depict the ± *SEM*.

The experiment used 40 rats (*n* = 8) with the experimental design detailed in the top panel of Figure 1. Twenty-four hours after a T2 transection, subjects were placed in the restraining tubes and tail electrodes were attached. All subjects were restrained for 30 min. During this period, subjects received 180, 360, 540, 720 or 900 FT tail shocks spaced 2 s apart. The onset of stimulation was delayed for subjects that received less than 900 FT shocks, to equate the interval between the last shock and the onset of testing. Subjects were then set-up for instrumental testing and received 30 min of response-contingent leg shock. Testing was conducted using either the left or right leg and this was counter-balanced across groups.

The effect of shock number on response duration is depicted in Figure 1A. Subjects given 360 or fewer shocks exhibited a learning deficit, replicating previous results (Baumbauer et al., 2008, 2009). Subjects given 540 or more shocks were able to learn when tested with controllable shock. An ANOVA revealed a main effect of shock number *F*_(4,35)_ = 13.15, *p* < 0.001. Also, the Time × Shock number interaction was significant, *F*_(29,1015)_ = 1.76, *p* < 0.001. *Post hoc* comparisons of the group means (Figure 1A) confirmed that subjects given 180 and 360 stimulations differed from those that received 540, 720 and 900 stimulations (*p* < 0.05). No other comparisons were significant (*p* > 0.05).

As in past studies, subjects that failed to learn exhibited the highest rate of responding (Figure 1B). An ANOVA showed that shock number affected the rate of responding, *F*_(4,35)_ = 5.80, *p* < 0.01. *Post hoc* comparisons of the group means showed that the groups that received 180 or 360 shocks differed from those that received 540 or more (*p* < 0.05). No other comparisons were significant (*p* > 0.05).

As previously reported, 180 fixed spaced shocks induced a learning impairment (Baumbauer et al., 2008, 2009). Continued exposure to fixed spaced stimulation eliminated this effect. The transition between these effects appears to occur after 360 shocks, with a minimum of 540. As observed in earlier studies, learning was accompanied by a reduction in the rate of responding (Grau et al., 1998, 2006). Indeed, the two groups that failed to learn (as indexed by an increase in flexion duration) exhibited the highest rate of responding. This is important because it shows that the failure to learn does not reflect a performance deficit. Because a similar inverse relation between response duration and number was observed in all of the subsequent experiments, and because the former provides a more straight-forward measure of learning (Grau et al., 1998), only response duration is presented in subsequent experiments.

### Experiment 2: Additional Stimulation Produces a Similar Result

Our assumption is that continued exposure to fixed spaced stimulation engages a qualitatively distinct process that transforms how spinal circuits function, restoring the capacity to learn and countering the maladaptive effects of variable shock (Baumbauer et al., 2008, 2009). It is implicitly assumed here that this transformation depends upon stimulus regularity—that only FT stimulation has a restorative effect. It could be argued, however, that the difference between regular and irregular stimulation is quantitative in nature, not qualitative. From this perspective, both VT and FT stimulation should have a non-monotonic effect, with a lower number of stimuli impairing plasticity and a higher number restoring the capacity for learning. For example, this could occur because stimuli separated by roughly 2 s are particularly effective at producing the protective effect. If it is then assumed that the relative effectiveness of this training interval is normally distributed, any distribution that includes these values (including VT stimulation) should produce a restorative effect, albeit less rapidly. We evaluated this possibility by comparing the effect of FT and VT stimulation when shock number is increased five fold (to 4,500 shocks).

**Figure 2:**
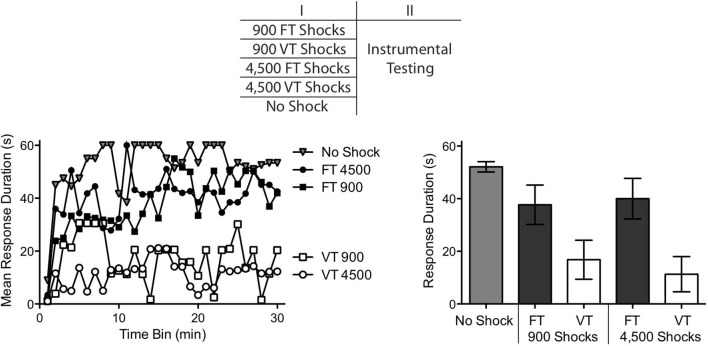
**Relative impact of 900–4,500 shocks, given in a fixed (FT) or variable (VT) manner, on instrumental learning.** Spinally transected rats received 900 FT or VT shocks, 4,500 FT or VT shocks, or no shock. Instrumental learning was then tested for 30 min. Subjects that received no shock or FT shock exhibited a progressive increase in response duration indicative of learning. Subjects that received 900–4,500 VT shocks failed to learn. The top panel depicts the design of the experiment. Performance over the 30 min test period is illustrated in the left panel and mean performance is depicted to the right. The error bars depict the ± *SEM*.

Forty rats underwent a spinal transection and were randomly assigned to one of five conditions (*n* = 8) as indicated at the top of Figure 2. A day after surgery, subjects were placed in the restraining tubes for a period of 150 min and had the tail electrodes attached. One group received no shock (Unshocked). The remaining subjects received either 900 or 4,500 tail shocks at 0.5 Hz on a FT or VT ISI. To equate the interval between the last shock and testing, subjects given 900 shocks received the stimulation during the last 30 min of restraint. Instrumental learning was then tested for 30 min as described above.

We found that FT and VT stimulation had divergent effects on spinal learning independent of whether subjects received 900 or 4,500 shocks (Figure 2). An ANOVA confirmed that the main effect of shock treatment was significant, *F*_(4,25)_ = 5.46, *p* < 0.005. There was also a significant effect of time, and Time × Shock Treatment interaction, both *F*’s > 1.52, *p* < 0.001. *Post hoc* comparisons of the group means showed that the two groups that received variable shock differed from the unshocked group and the two FT treated groups (*p* < 0.05). No other comparison was significant (*p* > 0.05).

If VT stimulation has a non-monotonic effect on learning, increasing shock number should transform how it affects spinal function and reinstate the capacity to learn. No evidence for this was obtained. Independent of the number of shocks presented (900 or 4,500), VT stimulation induced a learning impairment. This suggests that the difference between VT and FT stimulation is not simply quantitative in nature. Rather, exposure to FT stimulation appears to have a qualitatively distinct effect.

### Experiment 3: Shock Number, not the Duration of Exposure, is Critical

Experiment 1 showed that exposure to 540–900 fixed spaced shock re-established the capacity for instrumental learning, a hallmark of the FT effect. In subsequent experiments, we use this finding to explore the processes that underlie learning about time. To do so, we compare the effect of a sub-threshold level of stimulation (360 shocks) to the effect of 720 shocks. The latter value is used because it has been shown to have a lasting restorative effect and it is a multiple of 360, which simplifies our analyses of additivity.

In evaluating the effect of shock number, we have held shock frequency constant (at 0.5 Hz). As a result, shock number was confounded with duration of exposure. It is therefore possible that 360 and 720 shocks have distinct effects because the latter occurs over a period of time (24 min) that is twice as long. To address this possibility, we compared the effect of 360 vs. 720 shocks given over a period of 6, 12 or 24 min. If the emergence of the FT effect is tied to shock number, duration of exposure should not matter. Conversely, if the critical variable is the duration of exposure, 24 min of stimulation should reinstate the capacity to learn independent of whether subjects received 360 or 720 shocks.

**Figure 3:**
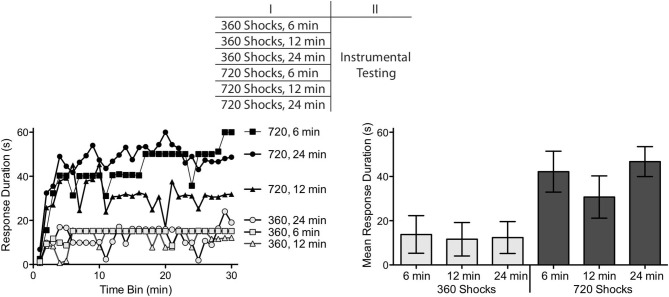
**Comparison of shock number and duration of exposure.** Subjects received 360 or 720 FT shocks over a period of 6, 12 or 24 min. Exposure to 360, but not 720, shocks produced a learning impairment independent of the duration of exposure. The top panel depicts the experimental design. The left panel illustrates the change in response duration observed over the 30 min of testing and the group means are provided to the right. The error bars depict the ± *SEM*.

This experiment used 48 subjects (*n* = 8). A day after the spinal transection, subjects were placed in restraining tubes and had tail electrodes attached as described above. They then received 360 or 720 tail shocks given over a period of 6, 12 or 24 min (Figure 3). All subjects were restrained for an equivalent period of time (24 min). To verify that stimulus exposure had a long-term effect on spinal function, instrumental testing was conducted 24 h later. Subjects were tested with 30 min of response-contingent shock as described above. The top panel of Figure 3 details the experimental design.

Exposure to 360 fixed spaced shocks induced a learning impairment independent of whether the shocks were presented over a period of 6 (1.0 Hz), 12 (0.5 Hz) or 24 min (0.25 Hz) (Figure 3). Conversely, rats given additional shocks (720) were able to learn independent of whether the stimuli were given over a period of 6 (2 Hz), 12 (1.0 Hz) or 24 min (0.5 Hz). An ANOVA revealed a main effect of shock number, *F*_(1,42)_ = 23.81, *p* < 0.001. Neither the main effect of session duration, nor the interaction between session duration and shock number, were significant, *F*s < 1.63, *p* < 0.5. *Post hoc* comparisons confirmed that the groups that received 360 stimulations were significantly different from the groups that received 720 stimulations (*p* < 0.05). No other group difference was significant (*p* > 0.05).

Within the frequency range tested, the emergence of the restorative effect of FT stimulation was determined by the number of shocks given, not the duration of exposure. Outside of these bounds, a different pattern could emerge. In part, this is because the learning impairment is most robust within a frequency range of 0.1–2.5 Hz (Crown et al., 2002). In addition, if the effect of FT stimulation reflects a form of learning, some trade-off would be expected between stimulus number and frequency, with spaced presentation being more effective than massed (Groves et al., 1969; Davis, 1970). From this perspective, higher frequency stimulation may have a restorative effect, but require a greater shock number.

### Experiment 4: Evidence of Savings Across Days

A hallmark of learning is the retention (memory) of information over time. For timing, a learning account assumes that a feature of the training is abstracted and stored, yielding some savings across days. From Experiment 1, we know that the emergence of the restorative effect requires more than 360 shocks. Here we assessed whether FT stimulation has a restorative effect when the additional (360) shocks are given 24 h later. We also tested whether it matters if the stimuli occur to the same, or a different, dermatome (tail vs. leg). If the FT effect reflects a form of spinal learning, that is centrally mediated, it should emerge when FT stimulation is given across days to distinct dermatomes.

**Figure 4:**
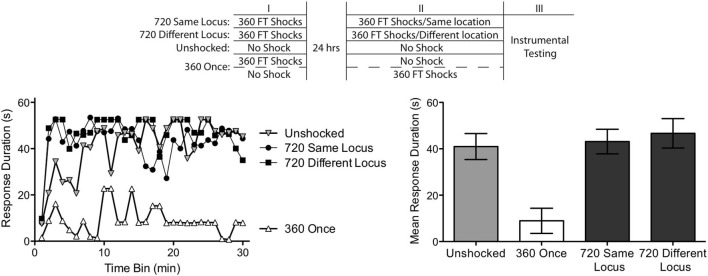
**Abstraction of regularity over time and shock locus.** Subjects received two shock treatments, starting a day after a spinal transection. One group (720 Same Locus) received 360 FT shocks on each day to the same location (the tail or leg). Another (720 Different Locus) received 360 FT shocks on each day, but on different dermatomes. A third group never received shock (Unshocked). The last group (360 Once) received a single bout of 360 FT shocks, either during the first or second session. The experimental design is depicted in the top panel. All subjects were then tested for 30 min with response-contingent shock applied to the untreated leg. As expected, unshocked rats exhibited an increase in response duration over the 30 min of testing (left panel). Exposure to a single bout of 360 FT shocks induced a learning impairment. Two bouts of FT shock eliminated the learning impairment and this was true independent of whether the shocks were given at the same or different locations across days. Mean performance, ± *SEM*, is illustrated in the right panel.

The experimental design is illustrated at the top of Figure 4. A day after spinal transection, two groups received 360 FT shocks at 0.5 Hz to the tail or leg. The next day, half of the subjects (720 Same Locus) received 360 FT shocks at 0.5 Hz to the same location (*n* = 8). The remaining subjects (*n* = 8) received shock to the other dermatome (720 Different Locus). A third group (Unshocked) was restrained, and had the electrodes attached on both days, but received no shock (*n* = 8). Subjects in a fourth group (360 Once) were also restrained on both days, but received just one bout of 360 FT shocks at 0.5 Hz. Half of these subjects (*n* = 4) were given shock on the first day while the remaining subjects (*n* = 4) received shock the next day. The site of shock treatment was counter-balanced across subjects. Immediately after the second shock treatment, instrumental learning was tested on the untreated hindleg as described above.

From the results of the previous experiments, we expected that subjects given just 360 fixed spaced shocks would exhibit a learning impairment independent of whether the stimuli occurred immediately before testing or 24 h earlier. Both treatments did, indeed, produce similar results, *F*_(1,6)_ < 1.0, *p* > 0.05. Given this, we collapsed the results across the two conditions to form a single condition (360 Once).

As shown in Figure 4, the Unshocked group learned whereas rats given a single session of 360 FT shocks (360 Once) did not. Most importantly, a second bout of 360 shocks given 24 h later reinstated the capacity to learn and this was true independent of whether the shocks were presented to the same (720 Same Locus) or different (720 Different Locus) dermatomes. An ANOVA revealed a significant main effect of shock condition, and a significant Time × Shock Treatment interaction *F*’s > 5.23, *p* < 0.05. *Post hoc* comparisons of the group means confirmed that the 360 Once group differed from all other groups (*p* < 0.05). No other differences approached significance (*p* > 0.05).

Subjects that received only 360 shocks exhibited a learning impairment. Exposure to additional FT stimulation reinstated the capacity to learn. Because the treatments were separated by 24 h, this implies that the initial bout of stimulation had an enduring effect, inducing a kind of memory that spanned the gap in time. Further, because this effect emerged when shocks were presented to distinct dermatomes, the results imply a form of spatial integration that must depend upon a central (spinal) system.

### Experiment 5: Spinal Neurons Encode an Index of Regularity, not the Specific Interval of Time

We have shown that two bouts of 360 FT shocks, separated by 24 h, can produce the FT effect. The implication is that a feature related to regularity is abstracted and stored during the initial bout of 360 shocks. One possibility is that spinal systems encode the particular interval (2 s) that separated the shocks, a process that we will refer to as *hard timing*. An alternative possibility is that spinal systems just encode that the incoming stimulus was regular in nature (an index of regularity). From this view, what matters is that both bouts are regular, not whether the ISI matches. We refer to this alternative as *soft timing*. To evaluate these alternatives, we again exposed subjects to two bouts of 360 shocks, separated by 24 h. However, in this experiment, the shocks within each bout were spaced either 0.5 s (2 Hz) or 2 s (0.5 Hz) apart. One group received shock at the same frequency across days (720 Same Hz) while another received shock at different frequencies (720 Different Hz). A third group (360 Once) received just a single bout of shock, at an ISI of 0.5 or 2 s, which should induce a learning impairment.

**Figure 5:**
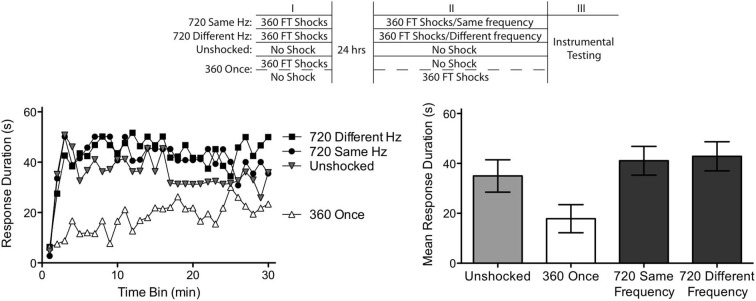
**Abstraction of regularity over time and shock frequency.** Subjects received two shock treatments, starting a day after a spinal transection. One group (720 Same Hz) received 360 FT shocks on each day at the same frequency (0.5 or 2 Hz). Another (720 Different Hz) received 360 FT shocks on each day, but on different frequencies. A third group never received shock (Unshocked). The last group (360 Once) received a single bout of 360 FT shocks, during either the first or second session. Half of these subjects received shock at 0.5 Hz while the remaining received shock at 2 Hz. This experimental design is detailed in the top panel. Subjects were then tested for 30 min with response-contingent shock applied to the untreated leg. As expected, unshocked rats exhibited an increase in response duration over the 30 min of testing (left panel). Exposure to a single bout of 360 FT shocks induced a learning impairment. Two bouts of FT shock eliminated the learning impairment and this was true independent of whether the shocks were given at the same or different frequencies across days. Mean performance, ± *SEM*, is illustrated in the right panel.

We conducted this experiment in two replications (*n* = 6) and used 48 rats. Because similar results were obtained across each replication, *F*_(2,30)_ = 1.03, *p* > 0.05, we collapsed the data across this variable. The experimental design is illustrated at the top of Figure 5. As in Experiment 4, subjects received two training sessions, beginning a day after surgery. The second session occurred 24 h after session 1. One group received 360 shocks on both days at the same frequency, either 0.5 of 2 Hz. For both conditions, subjects were restrained an equivalent period of time and shocks were presented to different dermatomes (leg or tail; with both stimulus frequency and dermatome counter-balanced across subjects). Because both conditions yielded similar results, *F*_(1,10)_ < 1.0, *p* > 0.05, we collapsed the data across these two conditions to form a single group (720 Same Hz). Other rats were treated the same but received shock at different frequencies across days (720 Different Hz). Again, both stimulation site and frequency were counter-balanced across days. A third group received a single bout of 360 shocks, given at an ISI of 0.5 or 2 s on Day 1 or 2. Because similar results were obtained across dermatome, ISI, and day, the data were collapsed to form a single group (360 Once). Finally, a fourth group was restrained an equivalent period of time, but remained unshocked. After the second treatment, subjects underwent 30 min of instrumental testing as described above.

Subjects given a single session of 360 shocks (360 Once) exhibited impaired learning (Figure 5). A second session of fixed spaced shock had a restorative effect independent of whether it was presented at the same or different frequency. An ANOVA confirmed that shock treatment had a significant effect, *F*_(2,33)_ = 5.40, *p* < 0.01. There was also a significant effect of time and Time × Shock Treatment interaction, both *F*’s > 1.72, *p* < 0.05. *Post hoc* comparisons of the group means showed that the groups that received one session of FT stimulation differed from the other two (*p* < 0.05). No other comparisons were significant (*p* > 0.05).

Our results provide further evidence of a savings effect, demonstrating that two bouts of regular stimulation can induce the FT effect when separated by 24 h. At issue was the nature of the memory laid down by the initial bout of stimulation. Do spinal systems abstract the specific interval between stimuli (hard timing) or simply an index of regularity (soft timing). Our results support the latter conclusion because a similar outcome was obtained irrespective of whether the shocks presented on Day 1 and 2 had the same or different frequencies.

## Discussion

Prior work had shown that the effect of fixed spaced shock varies as a function of shock number, with 180 shocks producing a learning impairment and 720 having a restorative effect (Baumbauer et al., 2008, 2009). Here we derived the shock number needed to transform the effect of stimulation, showing that a restorative effect emerges after 540 shocks (Experiment 1). We then showed that the effect of FT and VT stimulation remains stable if additional shocks (4,500) are given (Experiment 2). When 360 versus 720 FT shocks were given over 6, 12, or 24 min, we found that 360 shocks consistently produced a learning impairment whereas 720 did not (Experiment 3). This implies that the critical factor is shock number, not the duration of exposure. We then used this information to explore the nature of what is learned. It was assumed here that learning involves an incremental process that develops over the course of training. If this is true, an initial bout of FT stimulation (insufficient to restore learning) should have a lasting effect that fosters the development of the restorative effect when training is continued. We explored this possibility by administering two bouts of 360 FT shocks separated by 24 hrs (Experiment 4). As expected, a single bout of 360 shocks induced a learning impairment. When combined, the capacity for learning was restored, demonstrating a form of savings. Moreover, two bouts of shock yielded a restorative effect when applied to distinct dermatomes, implying a kind of central (spinal) integration. We then used this savings effect to explore the nature of the underlying memory. Here we introduced a distinction between hard and soft timing, suggesting that the former involves a memory for the temporal interval whereas the latter simply encodes that there was a period of regular stimulation. We explored this issue by applying two bouts of 360 FT shock and varying the frequency of stimulation. We found that FT stimulation had a restorative effect independent of whether the frequency of stimulation across bouts remained the same or differed (Experiment 5). This implies that spinal systems do not store the specific interval between shocks but rather encode that there was a period of regular stimulation.

Elsewhere, we have explored the mechanism that underlies the abstraction of regularity within the spinal cord (Lee et al., submitted). We reasoned that this could be accomplished by means of either an internal oscillator (a process that exhibits a pendulum-like regularity over time) or a timer (an hourglass-like device that decays over a fixed period). If an oscillator is at work, the occasional omission of a stimulus may have little effect; as long as subsequent shocks are presented in phase, the process could continue on at the same tempo. Supporting this, we found that randomly omitting up to half the shocks from a train of FT stimulation has no impact on the development of the restorative effect, provided the shocks remain in phase (Lee et al., submitted). In addition, we have observed that when FT stimulation is applied in combination with a drug cocktail that promotes spinally-mediated motor behavior (Strain et al., 2014), it can engage a rhythmic swinging of a the tail (pendulate tail) that occurs at the same frequency as the eliciting stimulus and continues for minutes after stimulation has ended (Strain et al., 2014). These observations suggest that regular stimulation engages a neural oscillator.

It has been recognized for many years that the lumbosacral spinal cord contains oscillators (central pattern generators, CPGs) that drive rhythmic behavior (e.g., stepping, scratching; Brown, 1911; Grillner, 1973; Grillner and Zangger, 1979; Rossignol et al., 1996, 2009; Kiehn and Kjaerulff, 1998; Marder and Bucher, 2001; Kiehn, 2006; Hultborn and Nielsen, 2007; Guertin, 2009; Rossignol and Frigon, 2011; Frigon, 2012). Research has further shown that the neural machinery needed to organize the pattern of leg movements lies within the lower lumbosacral (L3–S2) tissue (Cazalets et al., 1995; Magnuson et al., 1999, 2005), the same region required for spinally mediated instrumental learning (Liu et al., 2005). Interestingly, the neural system that sets the tempo of stepping is located rostrally, within the L1–L2 region. We have suggested that this CPG may mediate the abstraction of regularity. Supporting this, we have shown that surgically disconnecting the L1–L2 region from the lower lumbar spinal cord transforms how FT stimulation affects spinal function; when surgically disconnected from the L1–L2 region, an extended exposure to FT shock has a VT-like effect and induces a learning impairment (Lee et al., submitted).

These observations suggest that spinal timing depends upon the same neural systems that drive the tempo of stepping. The current work shows that engaging this oscillatory mechanism lays down a kind of memory that is preserved across days, allowing two sub-threshold bouts of stimulation to have an additive effect. What appears to underlie this cumulative effect is a process linked to the abstraction of regularity (e.g., that a neural oscillator was engaged), not the specific time interval. We further showed that what is important is not the duration that the oscillator is engaged, but rather the number of oscillations (stimuli). Similarly, Cha et al. (2007) found that increasing step number within a training session (from 100 to 1,000) had a lasting beneficial effect, increasing the quality of stepping over a range of treadmill speeds. Likewise, spinally transected animals that receive repeated bouts of step training across days exhibit a benefit from training, that restores the capacity to step on a treadmill across a range of treadmill speeds (de Leon et al., 1997, 1998; van de Crommert et al., 1998; Frigon and Gossard, 2009, 2010; van den Brand et al., 2012). Our work suggests that the emergence of these effect depends upon the number of regular steps taken each day rather than the duration of training. From this perspective, step training could allow spinal neurons to abstract a form of regularity, related to treadmill speed and step frequency. The current study implies that, within a training session, these factors should be kept constant (to allow for the abstraction of regularity), but across days different treadmill speeds (step rates) could be used. Introducing variability in treadmill speed within a session could undermine the long-term benefit of training, which may help to explain why some locomotor training paradigms appear less effective.

If step training and FT stimulation promote adaptive plasticity by engaging a common process, then the latter may be used to promote the former. Indeed, rhythmic stimulation to the tail or perineum is routinely used to promote stepping in spinally transected animals (Rossignol et al., 1996, 2009; Rossignol and Frigon, 2011; Alluin et al., 2015). FT stimulation and step training could also benefit adaptive plasticity for a common reason, for both may up-regulate the expression of BDNF (Gómez-Pinilla et al., 2002, 2007; Baumbauer et al., 2009; Huie et al., 2012b). Additionally, many forms of environmental perturbations (obstacles, immobilization, changes in load) that activate proprioceptive and afferent signals had been show to affect CPG output and cause adaptive changes, indicating that the spinal CPG is not only capable of adapting to environmental changes, but that these environmental changes can cause changes in behavior based on a form of temporal processing. Studies focused on the role of afferent feedback in modifying CPG activity have found that though phase durations and transitions are controlled by the spinal CPG, inputs from peripheral mechanoreceptors can alter the timing of the pattern of motor activation (Frigon and Gossard, 2009, 2010). When weak stimuli (simulating cutaneous tactile stimulation) or actual mechanical tactile stimulation was applied to the dorsal surface of the paw during the extension phase, activation of the extension muscles was markedly increased (Forssberg et al., 1975). Additionally, when an obstacle is placed on a treadmill during training after spinal cord injury, an increase in flexion is observed on subsequent steps and this hyperflexion elicited by cutaneous stimulation of the dorsal surface of the paw persists after the obstacle is removed (Nakada et al., 1994). This suggests a form of memory for the obstacle and implies that the behavior was timed to the swing phase. These results demonstrate that CPG entrainment can lead to a change in behavior due to learning about environmental stimuli.

Conversely, the adverse effect of VT stimulation appears to share commonalities with the processes that sensitize nociceptive neurons. Supporting this, we have shown that treatments that induce a central sensitization (e.g., peripheral application of the irritant capsaicin) impair instrumental learning (Ferguson et al., 2006; Hook et al., 2008). Further, both VT stimulation and capsaicin induce an enhanced mechanical reactivity (EMR) to von Frey stimuli applied to the plantar surface of the hind paw. These effects have been related to an up-regulation of the cytokine tumor necrosis factor (TNF; Huie et al., 2012a; Garraway et al., 2014). Just as FT stimulation opposes the adverse effect of VT shock, it also prevents and reverses both the learning impairment and EMR induced by capsaicin treatment (Baumbauer and Grau, 2011; Baumbauer et al., 2012). If FT stimulation and locomotor training engage common processes, the latter should also attenuate the development of neuropathic pain. Conversely, to the extent locomotor training involves a form of instrumental learning, we would anticipate that it too would be disrupted by peripheral inflammation. Recent observations support these hypothesized opponent relations (Hutchinson et al., 2004; Bouffard et al., 2014).

Taken together, our results imply that FT stimulation can have therapeutic value. This adds to the growing body of research demonstrating that both cutaneous and epidural stimulation can have a neuromodulatory effect (Harkema et al., 2011; Ferguson et al., 2012; Grau et al., 2014). What we have shown is that the effect of stimulation depends upon both behavioral control and temporal regularity—regular/controllable stimulation appears to promote adaptive plasticity by means of a BDNF-dependent process whereas irregular/unpredictable stimulation induces a form of maladaptive plasticity that inhibits spinal learning and enhances nociceptive reactivity through a TNF-dependent process (Grau et al., 2012, 2014). Our work suggests that these treatments can have a lasting effect. Further work is needed to evaluate the stimulus parameters that promote adaptive plasticity, the neurobiological mechanisms involved, and how these treatments can be translated to humans.

## Conflict of Interest Statement

The authors declare that the research was conducted in the absence of any commercial or financial relationships that could be construed as a potential conflict of interest.
